# Effects of varying Sm^3+^ concentration on the structure, morphology and photoluminescence properties of the BaAl_2_O_4_ /CaAl_2_O_4_/Ca_4_Al_6_O_13_/Ca_3_Al_2_O_6_:x% Sm^3+^ (0 ≤ x ≤ 1.9) mixed phases using citrate sol-gel method

**DOI:** 10.1016/j.heliyon.2022.e12573

**Published:** 2022-12-25

**Authors:** A. Bele, M.R. Mhlongo, L.F. Koao, T.E. Motaung, T.D. Malevu, T.T. Hlatshwayo, S. Mpelane, M. Mlambo, S.V. Motloung

**Affiliations:** a Department of Physics, Sefako Makgatho Health Science University, P. O. Box 94, Medunsa, 0204, South Africa; b Department of Physics, University of the Free State (Qwaqwa Campus), Private Bag X 13, Phuthaditjhaba, 9866, South Africa; c Department of Chemistry, Sefako Makgatho Health Science University, P. O. Box 65, Medunsa, 0204, South Africa; d Department of Chemistry, University of South Africa, P.O. Box 392, UNISA 0003, South Africa; e Department of Physics, University of Pretoria, Pretoria, 0002, South Africa; f Analytical Facility, University of Johannesburg, P.O. Box: 524, Auckland Park 2006, South Africa; g Health Platform, Advanced Materials Division, Mintek, 200 Malibongwe Drive, Randburg, South Africa; h Department of Chemical and Physical Sciences, Walter Sisulu University, Mthatha, 3886, South Africa

**Keywords:** Citrate sol-gel, Mixed phases, Luminescence, Sm^3+^

## Abstract

BaAl_2_O_4_/CaAl_2_O_4_/Ca_4_Al_6_O_13_/Ca_3_Al_2_O_6_:x% Sm^3+^ (0 ≤ x ≤ 1.9) (hereafter called BCCC:x% Sm^3+^) nanophosphors were successfully prepared by citrate sol-gel method. The structure, morphology and photoluminescence properties of the prepared nanophosphors were investigated. X-ray diffraction (XRD) indicated that the nanophosphors composed of the mixed phases of the hexagonal (CaAl_2_O_4_, BaAl_2_O_4_) and cubic (Ca_4_Al_6_O_13_, Ca_3_Al_2_O_6_) crystal structures. Scanning electron microscopy (SEM) revealed that doping influences the morphology of the prepared nanophosphor. High resolution transmission electron microscopy (HR-TEM) confirmed that the prepared phosphor particles are in the nanoscale range. Photoluminescence (PL) results showed emission peaks originating from the intrinsic defects within the BaAl_2_O_4_, CaAl_2_O_4_ and Sm^3+^ transitions. The optimum luminescence intensity was found at 0.7% Sm^3+^. Commission Internationale de l'éclairage (CIE) shows that the Sm^3+^ doped samples emitted the orange colour.

## 1 Introduction

In recent years, nanophosphor materials have received great attention from researchers around the globe due to their luminescence properties and this has led to the development of new light emitting devices (LEDs) [1]. Barium aluminate (BaAl_2_O_4_) is one of the multifunctional materials that can be used for the water purification, binders for ceramics and refractory, light-cumulative fluorescent materials, and afterglow phosphors [2, 3]. It has hexagonal structure with lattice parameters *a* = *b* = 10.4490 Å and *c* = 8.7930 Å [4]. It has the wide band gap energy (*E*_*g*_) ∼ 5.3 eV [5]. It is one of the best aluminate phosphors amongst the group of luminescence materials which has great efficiency, high quenching temperature, and high stability [6].

The other aluminate material that has been used in new applications in the field of advanced ceramics such as optical ceramics, catalyst support, flame detectors, dental cements and structural ceramics is calcium aluminate (CaAl_2_O_4_) [7]. CaAl_2_O_4_ possesses properties such as, compressive strength, splitting tensile strength, elastic modulus, stress-strain response, mass loss, compressive toughness and can easily be grown in crystalline form [8]. It has a wide *E*_*g*_ of 6 eV with hexagonal crystal structure and the lattice parameters *a = b =* 8.74 Å and *c =* 8.09 Å [9].

Reports have also focused on tetracalcium trialuminate (Ca_4_Al_6_O_13_) and tricalcium aluminate (Ca_3_Al_2_O_6_) [[10], [11]]. These calcium oxides are a special constituents of Portland cements. Ca_4_Al_6_O_13_ is a cubic indirect-gap semiconductor with *E*_*g*_ of 5.41 eV and the lattice parameters *a = b = c =* 8.86 Å [12] while Ca_3_Al_2_O_6_ has a wide band gap of 6.2 eV with cubic structure and the lattice parameters *a = b = c =* 7.624 Å [13, 14]. Generally, the crystalline powders depend on the method of preparation [15]. The synthesis of oxide phosphors has been achieved by a variety of methods such as the sol-gel method [16], co-precipitation [17], solid-state reaction [18], combustion [19] and hydrothermal [20]. In comparison with other methods, the sol-gel has advantages such as high homogeneity, safety, takes only few hours to complete, is environmentally friendly, produces nanopowder at low temperature, is very simple and very cheap [21]. Thus, the sol-gel method was employed in this study to synthesize BaAl_2_O_4/_CaAl_2_O_4_/Ca_4_Al_6_O_13_/Ca_3_Al_2_O_6_ (BCCC).

Recently, doping has been introduced to enhance the luminescence properties of the nanomaterials. In most cases, rare earth ions (RE^3+^) are introduced into the crystal structure of the host material. Among the RE^3+^ ions, Sm^3+^ have been studied due to its unique optical characteristics [22]. Shashikala et al. [23] reported the synthesis and photoluminescence (PL) studies on an orange-red colour emitting novel CaAl_2_O_4_:Sm^3+^ nanophosphor for light emitting diodes (LED) applications via combustion method. The results showed emission peaks centred at 564, 601 and 647 nm which were attributed to ^4^G_5/2_→^6^H_5/2_, ^4^G_5/2_→^6^H_7/2_ and ^4^G_5/2_→^6^H_9/2_ transitions of Sm^3+^, respectively.

Although investigations have been done on single phase host materials, a little has been explored on the mixed phases [24, 25, 26, 27]. Mixed phases might possibly result on the new and advanced phosphors materials with the combined properties of their bulk counterparts. For an example, Yuan et al. [25] showed that the mixed oxide ZnO/ZnAl_2_O_4_ has excellent stability and much higher photocatalytic activity than their bulk oxide counterparts. This study investigates the effect of varying the Sm^3+^ concentration on the structure, morphology, and photoluminescence properties of BCCC:x% Sm^3+^ mixed phases phosphor material. The optimum doping concentration was found at x = 0.7% Sm^3+^. Which resulted in tuning the emission colour from violet to orange. Therefore, this work will provide scope and add “new” knowledge to the development of new light-emitting materials that could be used in the fabrication of LED devices.

## 2 Experimental

### 2.1 Synthesis

BCCC:x% Sm^3+^(0 ≤ x ≤ 1.9) nanophosphors were successfully prepared by using citrate sol-gel method. The un-doped BCCC was prepared by dissolving Ba(NO_3_)_3_.6H_2_O (98%), Ca(NO_3_)_3_.6H_2_O (98%), Al(NO_3_)_3_.9H_2_O (98%) and citric acid (CA) C_8_H_8_O_7_.H_2_O (99%) in deionized water. Similar procedure was followed for the preparation of BaAl_2_O_4_ and CaAl_2_O_4_. The doped samples were prepared by varying x% Sm^3+^ (0 ≤ x ≤ 1.9) into the prepared un-doped sample nanophosphor. The sols stoichiometric molar ratio of Ba:Ca:Al and Ba:Ca:CA was found to be 1:1:2 and 1:1:0.75, respectively. The solution was constantly stirred using a magnetic stirrer at a constant temperature of 80 °C until the gels were formed. The gels were left overnight for more gelling. The gels were annealed in a furnace at 1000 °C for 2 h. This was done to ensure formability of the as synthesized samples. The solid products were grinded using mortar and pestle to form powder samples. The powder samples were then analysed using different techniques.

### 2.2 Characterization

The crystal structure of the prepared samples was characterized by Bruker D8-Advanced powder XRD with a Cu-Kα (1.5405 Å). The presence of multi-phases were identified using X'Pert Highscore plus software and the relative phase amounts (weight %) were estimated using the Rietveld method. The surface morphology, elementary composition and particle distribution of the prepared phosphors was investigated using a Zeiss Supra 55 scanning electron microscope (SEM) coupled with an energy dispersive X-ray spectroscopy (EDS). The nanorods images and SAED patterns were obtained using High-resolution transmission electron microscopy (HR-TEM) JEM-2100 equipped SAED operated at 200 keV accelerated voltage. Luminescence spectra and the lifetime measurements were performed at room temperature using the Hitachi F-7000 fluorescence spectrophotometer.

## 3 Results and discussion

### 3.1 X-ray diffraction (XRD)

**Figure 1:**
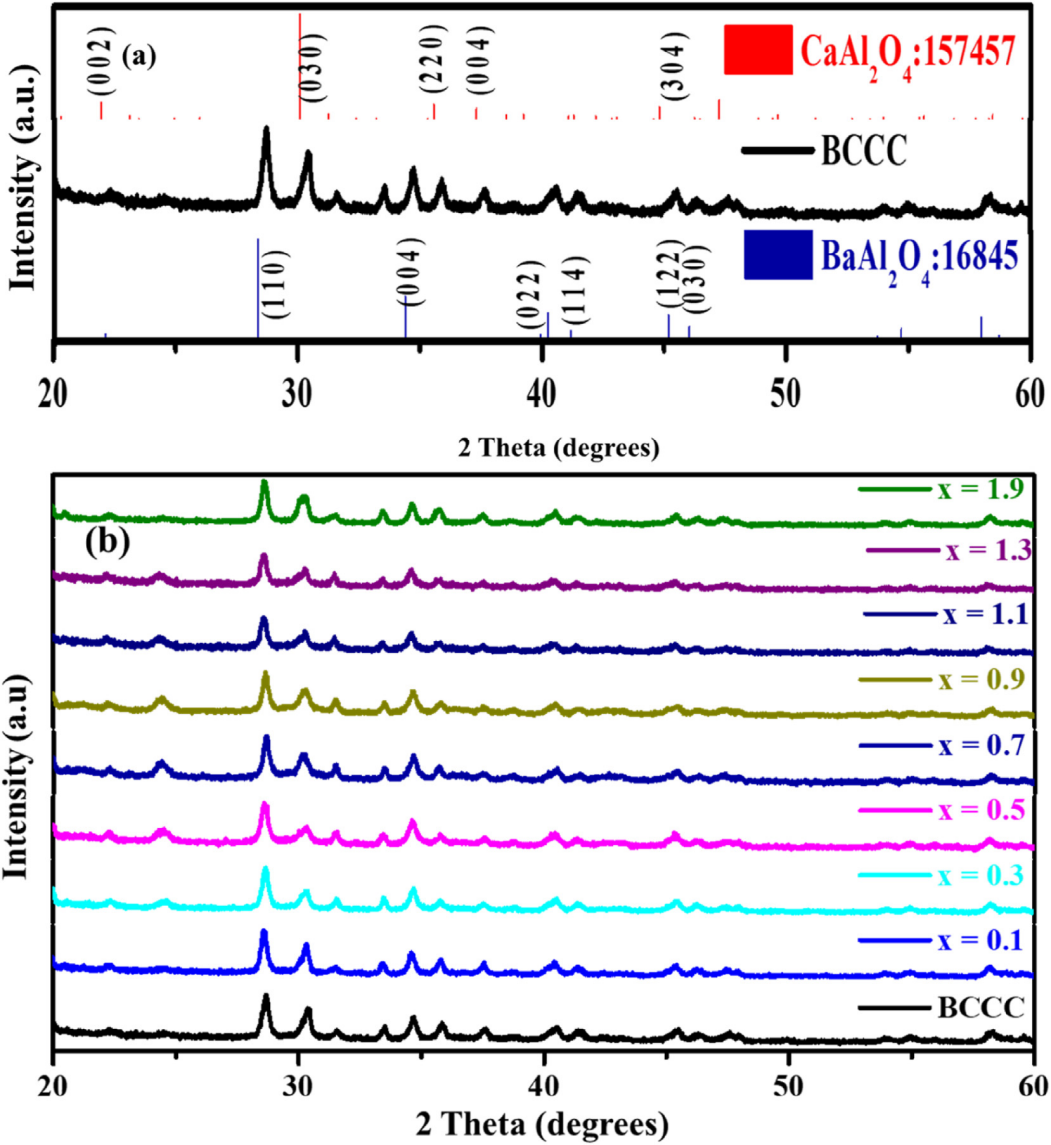
The X-ray patterns of the (a) BCCC and (b) BCCC:x% Sm^3+^ (0 ≤ x ≤ 1.9) samples.

XRD patterns for the BCCC and BCCC:x% Sm^3+^(0 ≤ x ≤ 1.9) are shown in Figure 1. Figure 1 (a) indicate that the nanophosphors consist of the mixed phases of the hexagonal (CaAl_2_O_4_, BaAl_2_O_4_) and Cubic (Ca_4_Al_6_O_13_, Ca_3_Al_2_O_6_) crystal structures. These structures matched ICSD cards 157457, 16845, 16177 and 151369, respectively. Figure 1 (b) shows the patterns of BCCC:x% Sm^3+^(0 ≤ x ≤ 1.9) samples and the results confirmed similar diffraction patterns to the one of the BCCC sample, which suggest that doping does not affect the crystal structures of the mixed phases. Similar results have been reported by Shashikala et al. [23], and Zhang et al. [28].

**Figure 2:**
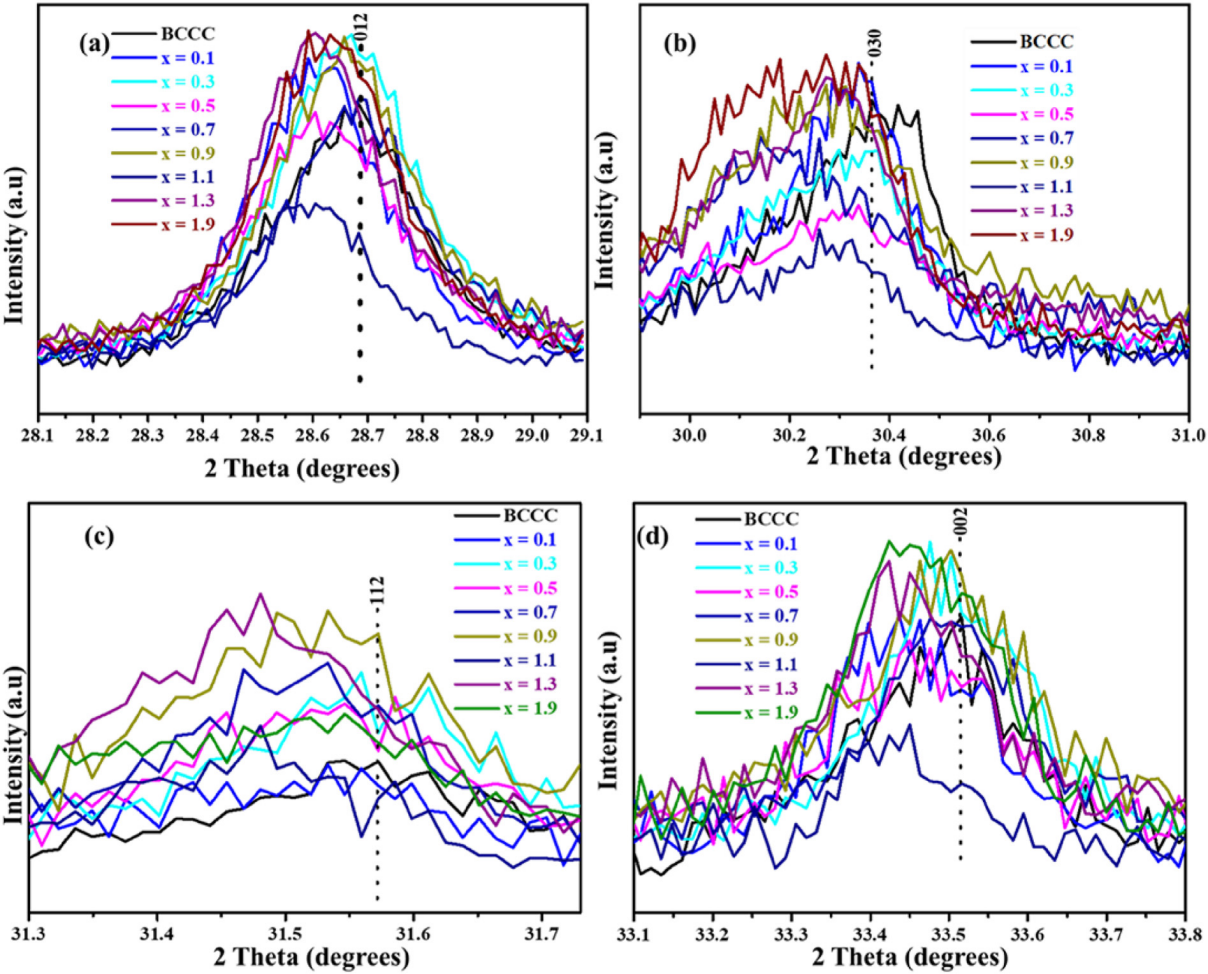
The analysis of the most intense diffraction peaks for (a) (012) of BaAl_2_O_4_ (030) of CaAl_2_O_4_ (112) of Ca_4_Al_6_O_13_ and (002) of Ca_3_Al_2_O_6_.

Figure 2 (a)–(d) shows the analysis of the most intense peaks (012) (030) (112) and (002) of the BaAl_2_O_4_, CaAl_2_O_4_, Ca_4_Al_6_O_13_ and Ca_3_Al_2_O_6_ phases, respectively. Generally, the results show that there is a shift of diffraction peaks towards the lower angles when doping with Sm^3+^. The peak shift to the lower diffraction angle is attributed to the increase in lattice parameters [29]. The lattice parameter increase is attributed to the replacement of smaller atoms with bigger atom in crystal lattices of individual phases. The incorporation of Sm^3+^ to the phases within the BCCC lattice is likely to be by substitution of either Ba^2+^ (1.34 Å) [30], Ca^2+^(1.12 Å) [31] or Al^3+^ (0.53 Å) [30]. Thus, we propose that Sm^3+^ (1.079 Å) [32] is possibly replacing the Al^3+^ in a lattice of each phase present in BCCC. Thus, these results suggest that the Sm^3+^ was successfully incorporated into crystal structure of the BCCC mixed phase. The phase quantification of the individual phase is shown in Table 1. The phase quantification revealed that in all samples, the CaAl_2_O_4_ phase is the highest in all samples.

**Table 1:** Phase quantification of the BaAl_2_O_4_, CaAl_2_O_4_, Ca_4_Al_6_O_13_ and Ca_3_Al_2_O_6_ mixed phases.

Sample ID	CaAl_2_O_4_ (%)	BaAl_2_O_4_ (%)	Ca_4_Al_6_O_13_ (%)	Ca_3_Al_2_O_6_ (%)
BCCC	58.4	22.5	9.4	9.7
x = 0.1	58.4	23.0	8.5	10.1
x = 0.3	45.1	24.6	21.3	9.0
x = 0.5	38.2	22.9	30.7	8.2
x = 0.7	45.9	18.5	28.9	6.7
x = 0.9	44.6	18.6	29.5	7.4
x = 1.1	42.1	22.5	29.3	6.1
x = 1.3	47.3	20.8	24.5	7.4
x = 1.9	61.0	21.5	8.3	9.2

The lattice parameters for the hexagonal (CaAl_2_O_4_, BaAl_2_O_4_) and cubic (Ca_4_Al_6_O_13_, Ca_3_Al_2_O_6_) were calculated from Eqs. (1) and (2),

(1) $$d_{hkl}=\frac{a}{\sqrt{h^{2}+k^{2}+l^{2}}}$$

(2) $$d_{hkl}=\frac{1}{\sqrt{\frac{4\left(h^{2}+k^{2}+hk\right)}{{3a}^{2}}+\left(\frac{l^{2}}{c^{2}}\right)}}$$

where, a,b and c are the lattice parameter, *d* is the interplanar distance and *hkl* are the Miller indices [33]. The lattice parameters of the un-doped (BCCC) mixed phases sample are presented in Table 2. The average lattice parameters for the hexagonal CaAl_2_O_4_ were estimated to be *a = b =* 8.74 Å and *c =* 8.09 Å, while for hexagonal BaAl_2_O_4_ were *a = b =* 5.21 Å and *c =* 8.76 Å, which is similar to the reported values in Ref. [34, 35]. The lattice parameters for the cubic (Ca_4_Al_6_O_13_ and Ca_3_Al_2_O_6_) structures were respectively estimated to be *a = b = c =* 8.86 Å and 7.624 Å, which is comparable to the reported values in Ref. [12, 14].

**Table 2:** Estimated lattice parameters of the un-doped mixed phases sample.

Lattice Parameter (Å)	BaAl_2_O_4_ (012)	CaAl_2_O_4_ (030)	Ca_4_Al_6_O_13_ (112)	Ca_3_Al_2_O_6_ (002)
a	5.21	8.74	8.86	7.62
b	5.21	8.74	8.86	7.62
c	8.76	8.09	8.86	7.62

The crystallite size (*D*) of the un-doped and BCCC:x% Sm^3+^ were estimated from most prominent diffraction peaks (012) (030) (112), and (002) using the Scherer's formula given in Eq. (3) [36].

(3) $$D=\frac{0.9\lambda }{\beta \;\mathrm{COS}\;\theta \;}$$

where *D* is the crystallite size (nm), λ is the radiation wavelength (0.15406 nm), *β* is the full width at half maximum (FWHM) (radians) and θ is the angle of diffraction (degrees). The estimated crystallites sizes are presented in Table 3. The results show that doping concentration influences the crystal size of the prepared nanophosphors.

**Table 3:** Estimated crystallite sizes (nm) of the prepared mixed phosphors.

Sample ID	BaAl_2_O_4_ (012)	CaAl_2_O_4_ (030)	Ca_4_Al_6_O_13_ (112)	Ca_3_Al_2_O_6_ (002)
BCCC	34	32	53	44
x = 0.1	38	37	43	47
x = 0.3	36	28	54	51
x = 0.5	34	24	45	44
x = 0.7	37	26	55	58
x = 0.9	37	26	35	49
x = 1.1	39	25	43	70
x = 1.3	40	28	46	56
x = 1.9	38	23	22	51

### 3.2 Energy dispersive X-ray spectroscopy (EDS)

**Figure 3:**
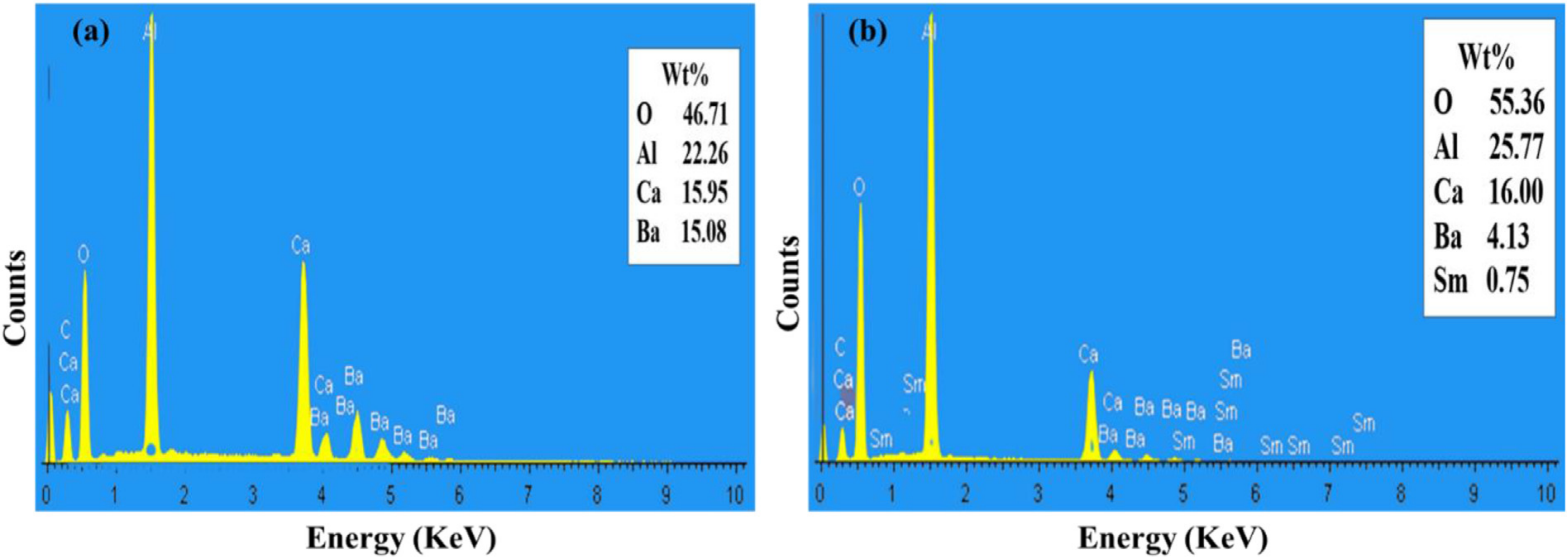
The EDS spectrum of the (a) BCCC sample and (b) x = 0.7% samples.

**Figure 4:**
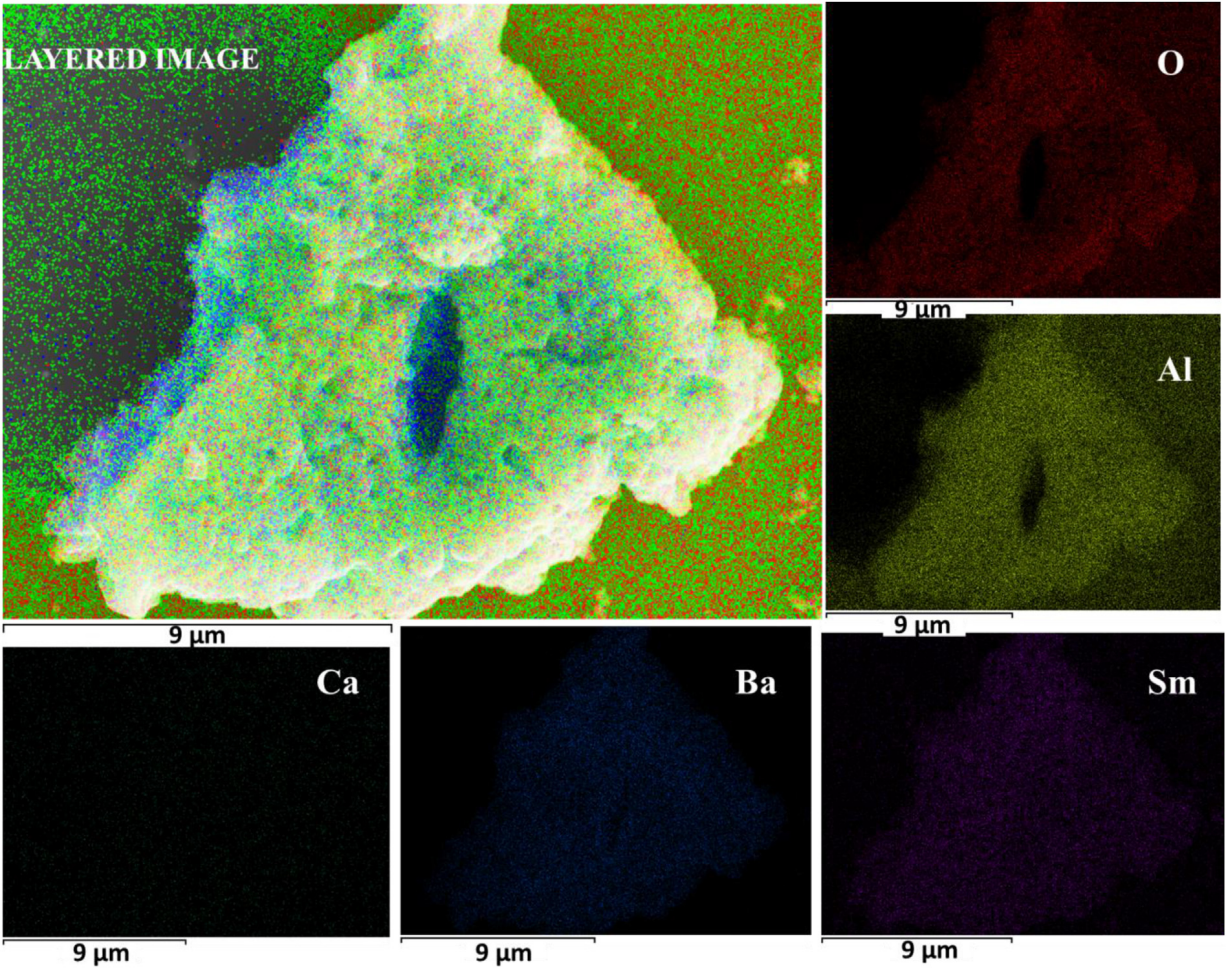
The EDS elemental map of the x = 0.7% sample.

Figure 3 (a) shows the EDS spectra of the BCCC sample. The results affirm the presence of the Ba, Ca, Al, and O as expected. Figure 3 (b) shows the x = 0.7% sample, which confirmed the presence of the elementary compositions of the BCCC and Sm. The presence of carbon (C) peak is due to carbon tape which was used to coat the sample holder during the analysis. No external impurities were observed. The EDS elemental map of the x = 0.7% is shown in Figure 4. The results indicate that the Ba, Ca, Al, O and Sm^3+^ were evenly distributed on the surface.

### 3.3 Scanning electron microscope (SEM)

**Figure 5:**
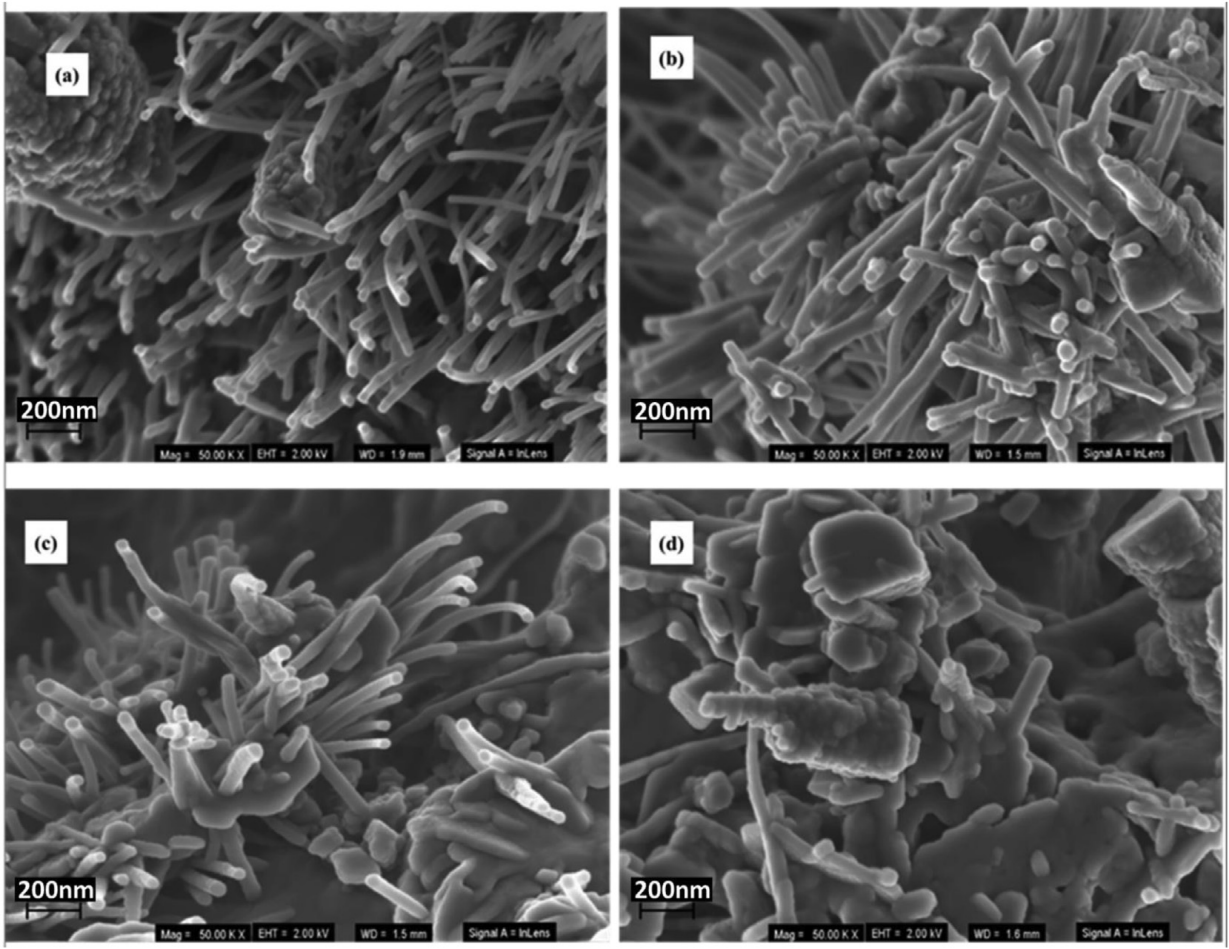
SEM images of the (a) BCCC (b) x = 0.1% (c) x = 0.7% and (d) x = 1.9% samples.

Morphological aspect of the selected nanophosphor samples was analyzed with SEM technique as given in Figure 5. Figure 5 (a) shows the BCCC sample, and the results reveal the presence of the nano-rods that are packed randomly with additional irregular particles. Similar morphology is observed for the samples doped with the x = 0.1% in Figure 5 (b) and 0.7% in Figure 5 (c). Figure 5 (d) shows the x = 1.9% sample, and the results clearly reveal the morphological change to the mixture of nano-rods and irregular particles. These results suggest that at higher Sm^3+^ doping concentration, denser irregular particles are formed with lower population of the rods structures. It is very clear that the Sm^3+^ concentration influences the morphology of the prepared samples.

### 3.4 High-resolution transmission electron microscopy (HR-TEM)

**Figure 6:**
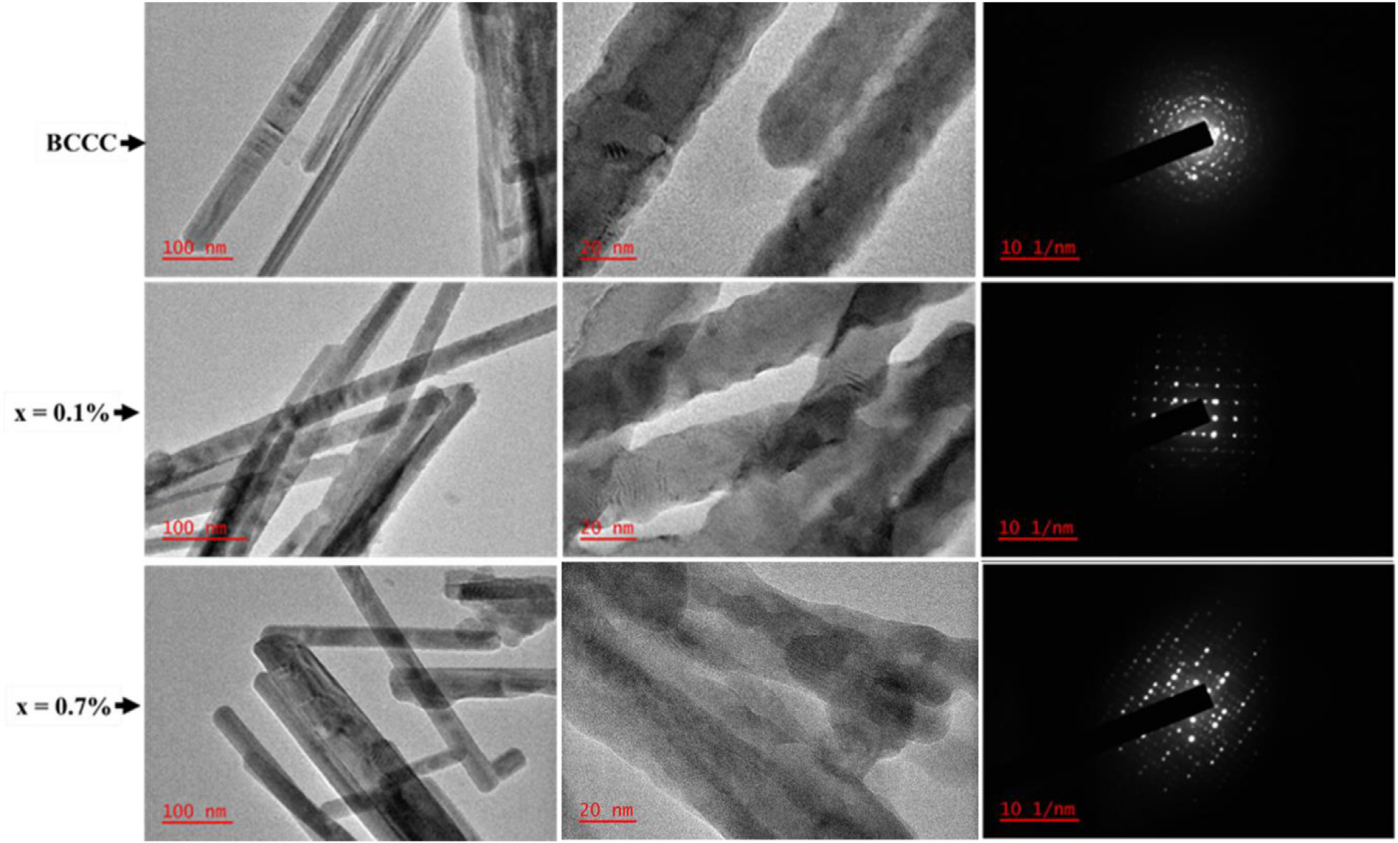
HR-TEM and SAED images of the BCCC, x = 0.1%, and x = 0.7%.

Figure 6 shows the HR-TEM images together with their respective SAED patterns. The BCCC, x = 0.1%, x = 0.7% reveal the nano-rod nature of the samples. The middle column shows the higher magnification of respective samples, and the lattice fringes can be seen, which confirms the crystalline nature of the samples. The SAED image of the samples also confirmed that the samples are highly crystalline. The SAED images could not be indexed due to samples undergoing phase transition under the HR-TEM beam. This phenomenon is well known for oxides [37] and maybe this can also be considered as another proof that the samples consist of mixed phases.

### 3.5 Photoluminescence (PL) spectroscopy

**Figure 7:**
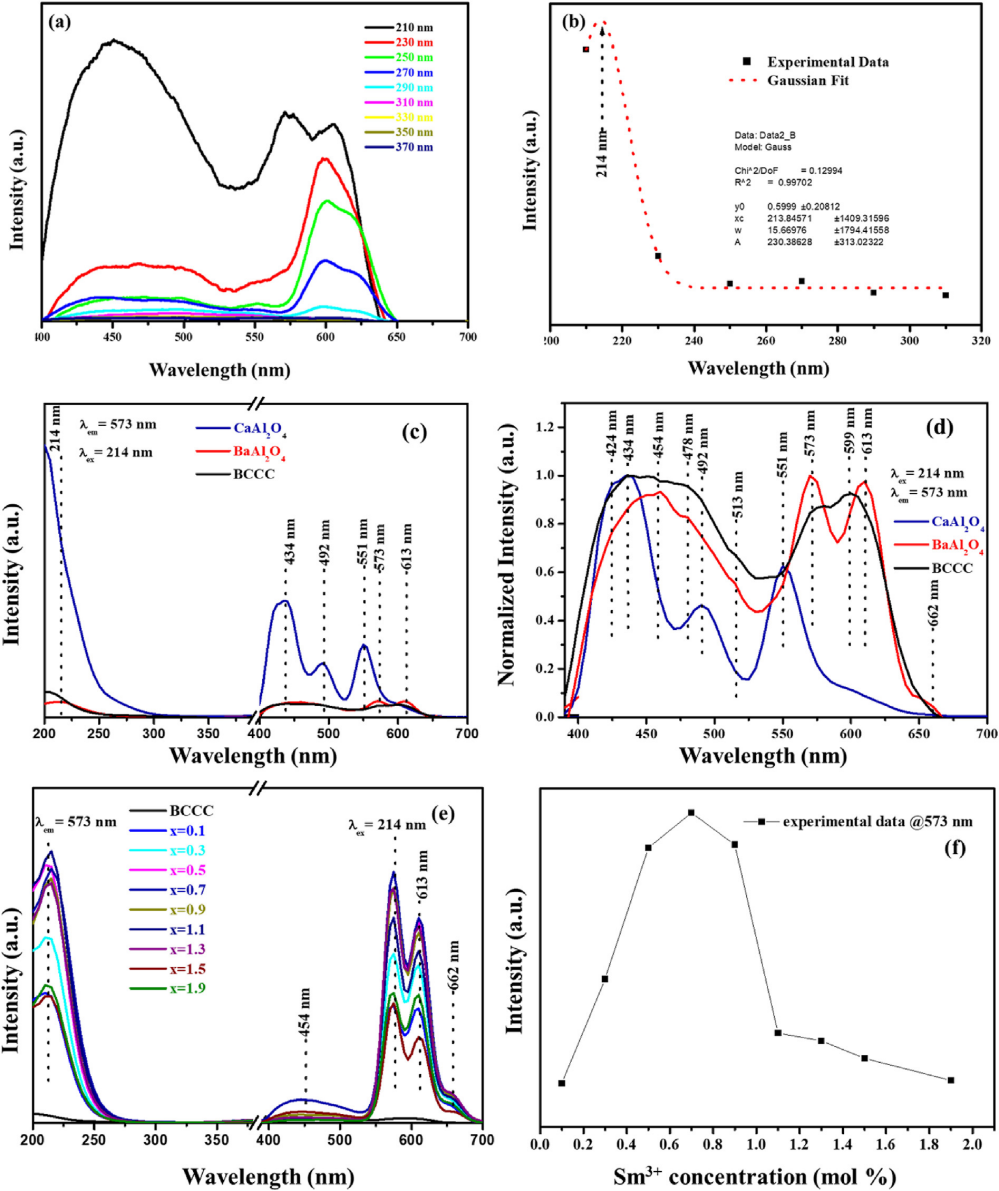
(a) BCCC excitation spectrum at different wavelengths, (b) Optimum excitation (Gaussian fit) (c) Emission spectrum of un-doped samples (BaAl_2_O_4_, CaAl_2_O_4_, and BCCC) (d) normalized spectrum of figure (c), (e) excitation and emission spectrum of un-doped and BCCC:x% Sm^3+^ (0 ≤ x ≤ 1.9), and (f) emission intensity at 573 nm as a function of Sm^3+^ concentration.

Figure 7 (a) shows the PL emission spectra of the BCCC sample excited at different wavelength. Figure 7 (b) shows the emission intensity as a function of the excitation wavelength and the Gaussian fit results revealed that the optimum excitation wavelength is 214 nm. This excitation is attributed to the band-to-band excitation of the BaAl_2_O_4_ [38]. Figure 7 (c) shows the excitation and emission spectra of the CaAl_2_O_4_, BaAl_2_O_4_, and BCCC samples. The emission bands were observed at 434, 492, 551, 573 and 613 nm Figure 7 (d) shows the normalize emission spectra of Figure 7 (c), which shows the additional emission peaks at 424, 454, 478, 514, 599 and 662 nm. The emission peaks at 424, 434, 492, 551 and 599 nm are attributed to the intrinsic defects in CaAl_2_O_4_ [39]. The results suggest the presence of trap centers in CaAl_2_O_4_ such as $V_{O}^{2+}\text{.}$ Emission peaks at 454, 478, 513, 573, 613 and 662 nm are attributed to the intrinsic defects within BaAl_2_O_4_ such as oxygen vacancies (*V*_*O*_) and Ba vacancies (*V*_*Ba*_) [40]. This also suggest that there are luminescence active traps located at different energy levels within BaAl_2_O_4_. Figure 7 (e) shows the excitation and emission spectra of the doped samples. The results show emission peaks also at 454, 573 and 613 nm with an additional peak at 662 nm. The emission peaks at 573, 613 and 662 nm can also be attributed to ^4^G_5/2_ → ^6^H_5/2_, ^4^G_5/2_ → ^6^H_7/2_ and ^4^G_5/2_ → ^6^H_9/2_ transitions of Sm^3+^, respectively [41]. The emission intensity as a function of Sm^3+^ concentration is shown in Figure 7 (f), which shows that the optimum concentration is at x = 0.7%.

**Figure 8:**
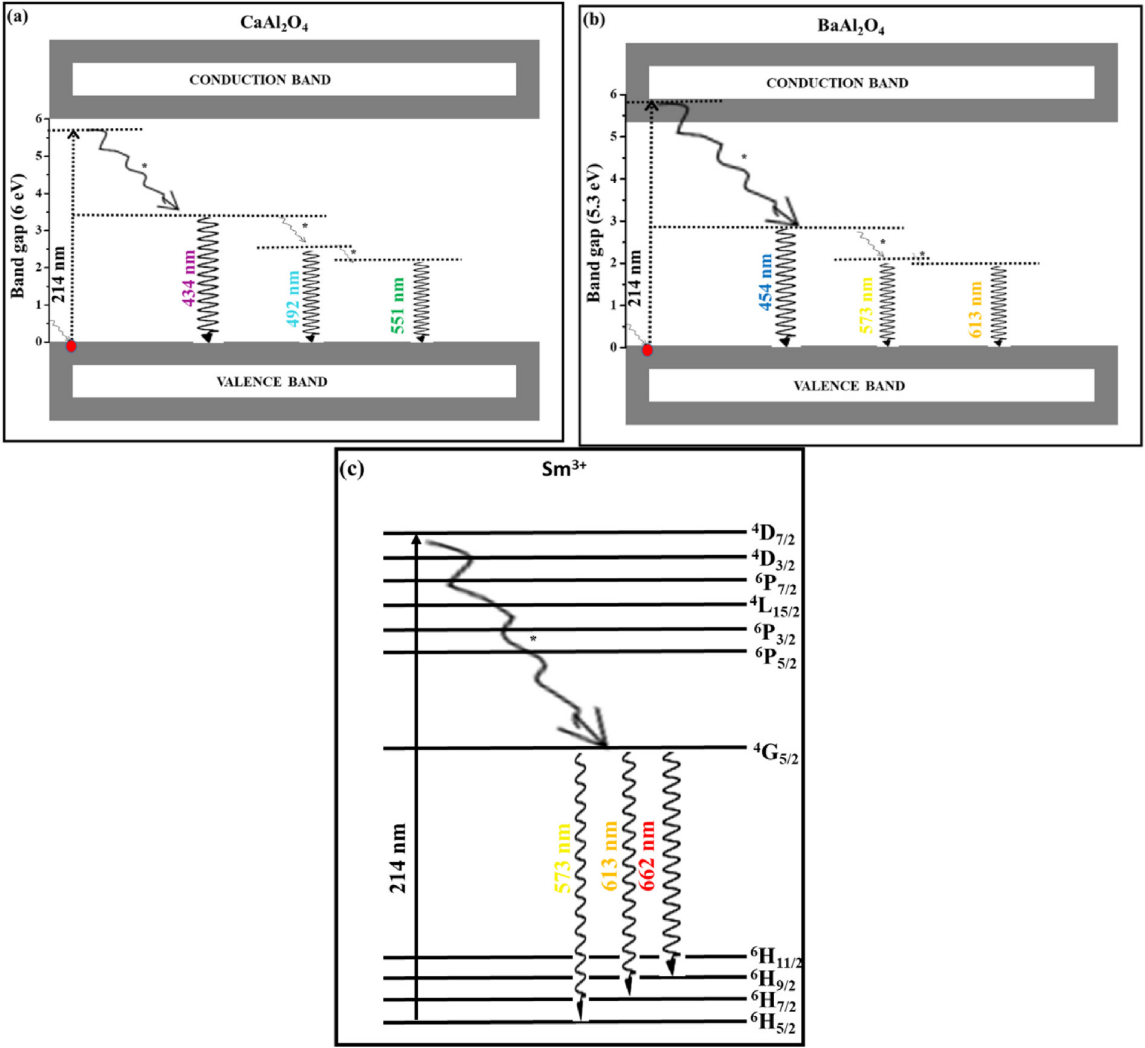
The proposed excitation and emission pathway mechanism from the (a) CaAl_2_O_4_, (b) BaAl_2_O_4_, and (c) Sm^3+^.

Figure 8 (a)–(c) shows the proposed emission pathways. Emission occurs when electron absorb enough energy and get excited from the valence band (VB) to the conduction band (CB). Then after excitation they lose energy and returns to the VB. However, after losing energy some of the electrons are de-excited by non-radiative relaxation denoted by (∗) and trapped within the defect's centres present inside the BaAl_2_O_4_ and CaAl_2_O_4_ material.

**Figure 9:**
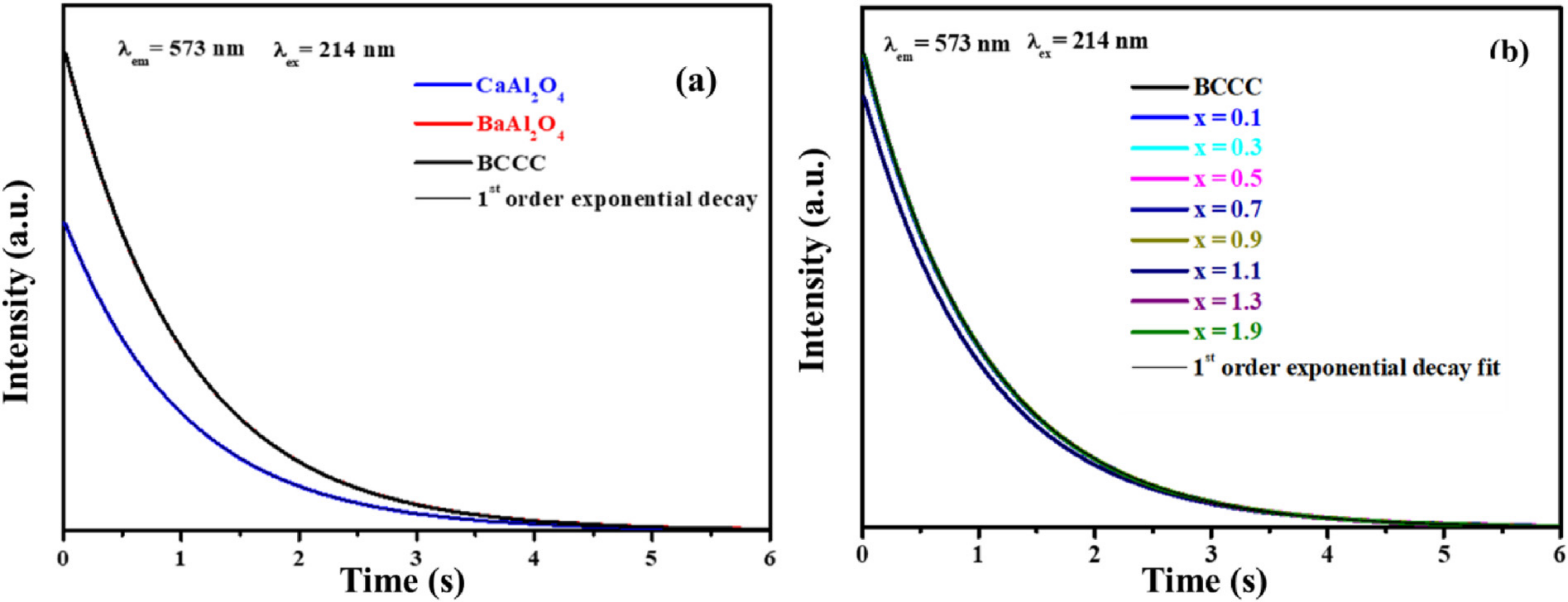
The exponential decay curves of the 573 nm emission for the (a) BCCC, CaAl_2_O_4_, BaAl_2_O_4_, (b) BCCC: x% Sm^3+^ (0 ≤ x ≤ 1.9) series.

Figure 9 presents he lifetime measurements (at room temperature) taken at 214 nm excitation and 573 nm Figure 9 (a) shows the exponential decay curves of CaAl_2_O_4_, BaAl_2_O_4_ and BCCC. Figure 9 (b) shows the exponential decay curves for BCCC: x% Sm^3+^ (0 ≤ x ≤ 1.9) series. The decay curves were fitted with 1^st^ order exponential decay Eq. (4) [42].

(4) I(t)=Aexp(−t/τ)

where *I* represent the phosphorescent intensity, A is the fitting parameter which contributes to the decay component, *t* is the time of measurement and τ is the decay time. The obtained values for the decay time and the fitting parameter are shown in Table 4. The results show that doping with Sm^3+^ concentration does not affect the afterglow mechanism. The results also show a longer lifetime. The longer lifetime could be attributed to the phases within the BCCC [43], which are due to the defects present within these individual phases [44]. In comparison to the un-doped BCCC sample, the lifetime induced by the Sm^3+^ is quite short ∼0.01 s on average. Thus, this clearly indicates that the long lifetime of the samples must be attributed to the matrix within the BCCC mixed phases. The results also confirm that the Sm^3+^ forms a new type of trap within these phases, which are luminescence active although their lifetimes are quite short.

**Table 4:** Summary of sample identification fitting parameter, decay time and CIE coordinates.

Sample ID	A	τ(ms)	CIE (x; y)
BCCC	10142.8 ± 1.9	1025.6 ± 0.2	(0.308; 0.298)
x = 0.1	10082.8 ± 0.2	1025.8 ± 0.2	(0.536; 0.433)
x = 0.3	10165.3 ± 0.1	1026.8 ± 0.8	(0.541; 0.435)
x = 0.5	10165.3 ± 0.1	1026.8 ± 0.2	(0.541; 0.440)
x = 0.7	10168.1 ± 1.6	1028.9 ± 0.2	(0.502; 0.413)
x = 0.9	10168.7 ± 1.1	1027.5 ± 0.2	(0.530; 0.434)
x = 1.1	9241.5 ± 0.4	1026.2 ± 0.2	(0.534; 0.440)
x = 1.3	10179.8 ± 3.8	1027.3 ± 0.2	(0.535; 0.440)
x = 1.9	10156.5 ± 1.2	1025.9 ± 0.2	(0.541; 0.436)

**Figure 10:**
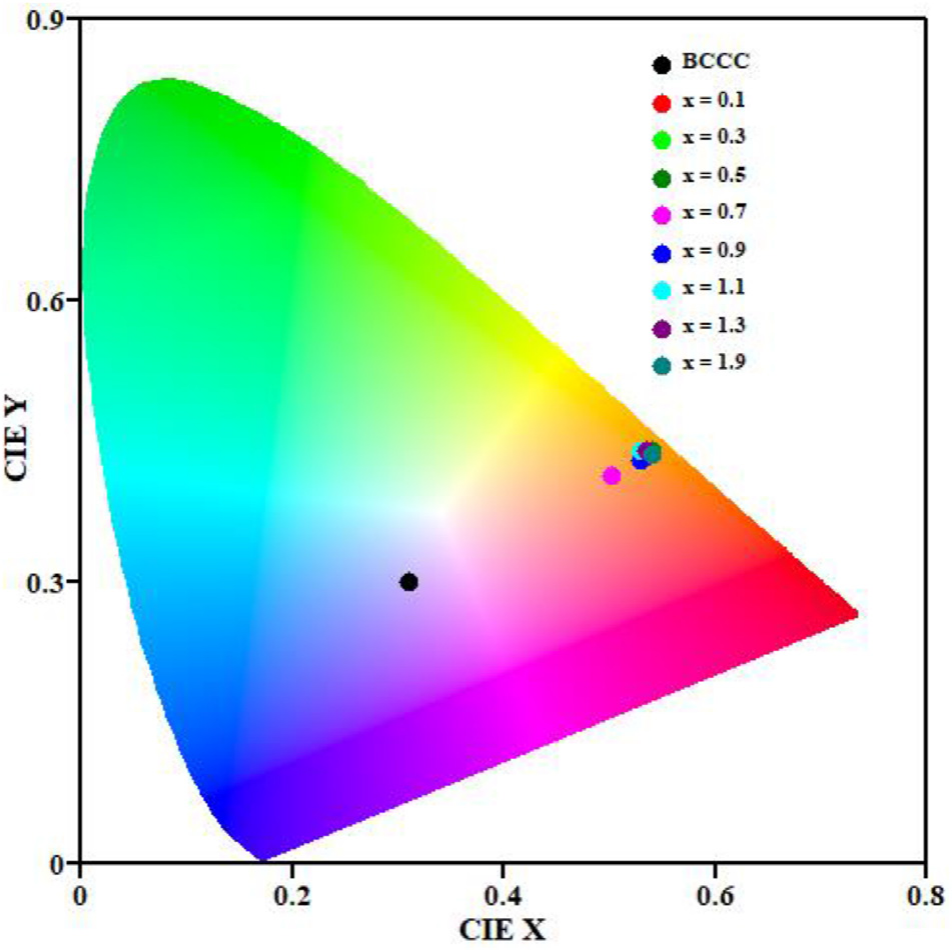
CIE diagram of the BCCC: x% Sm3+ (0 ≤ x ≤ 1.9) series.

Figure 10 shows the Commission Internationale de l'éclairage (CIE) colour chromaticity coordinates of the prepared samples, which was obtained using CIE coordinate calculator software. The colour coordinates are shown in Table 4. The results show that varying the Sm^3+^ concentration tuned the emission colour from violet to orange. These results also confirm the orange PL emission at 573 nm.

## 4 Conclusion

BCCC:x% Sm^3+^ (0 ≤ x ≤ 1.9) nanophosphors were successfully prepared using citrate sol-gel method. XRD suggested that Sm^3+^ doping does not change the crystal lattice of BCCC. SEM showed doping with Sm^3+^ alters the morphology of the prepared mixed nanophosphor. EDS confirmed all the expected elementary composition. PL results showed emission peaks at 424, 434, 454, 478, 492, 513, 551, 599, 573, 613 and 662 nm. Peaks at 424, 434, 492, 551 and 599 nm were attributed to the defects within CaAl_2_O_4_ and peaks at 454, 478, 513, 573, 613 and 662 nm were attributed to the defects in BaAl_2_O_4_. The doped samples showed the peaks at 573 nm, 613 nm and 662 nm which were attributed to ^4^G_5/2_ → ^6^H_5/2_, ^4^G_5/2_ → ^6^H_7/2_ and ^4^G_5/2_ → ^6^H_9/2_ transitions of Sm^3+^. The optimum doping concentration for Sm^3+^ was found to be x = 0.7%. The long lifetimes of the samples were attributed to the matrix within the BCCC mixed phases, while the Sm^3+^ contributed a very short lifetime. Commission Internationale de l'éclairage (CIE) showed that emission colour can be tuned from violet to orange by varying Sm^3+^ concentration.

## Declarations

### Author contribution statement

A. Bele: Conceived and designed the experiments; Performed the experiments; Analyzed and interpreted the data; Contributed reagents, materials, analysis tools or data; Wrote the paper.

M.R. Mhlongo, L.F. Koao: Conceived and designed the experiments; Analyzed and interpreted the data; Contributed reagents, materials, analysis tools or data.

T.E. Motaung, T. D. Malevu, T.T. Hlatshwayo, S. Mpelane, M. Mlambo: Analyzed and interpreted the data; Contributed reagents, materials, analysis tools or data.

S.V. Motloung: Conceived and designed the experiments; Analyzed and interpreted the data; Contributed reagents, materials, analysis tools or data; Wrote the paper.

### Funding statement

This work is supported by the South African National Research Foundation (NRF) Thuthuka programme (fund number: UID 13947), NRF incentive funding for rated researchers (IPRR) (Grant No: 114924).

### Data availability statement

Data will be made available on request.

### Declaration of interests statement

The authors declare no conflict of interest.

### Additional information

No additional information is available for this paper.
